# Ionizing Radiation Monitoring Technology at the Verge of Internet of Things

**DOI:** 10.3390/s21227629

**Published:** 2021-11-17

**Authors:** Muhammad Ikmal Ahmad, Mohd Hafizi Ab. Rahim, Rosdiadee Nordin, Faizal Mohamed, Asma’ Abu-Samah, Nor Fadzilah Abdullah

**Affiliations:** 1 Nuclear Science Programme, Department of Applied Physics, Faculty of Science and Technology, Universiti Kebangsaan Malaysia, Bangi 43600, Selangor, Malaysia; m.ikmalahmad@yahoo.com (M.I.A.); faizalm@ukm.edu.my (F.M.); 2 Technical Support Division, Atomic Energy Licensing Board (AELB), Jalan Dengkil, Batu 24, Dengkil 43000, Selangor, Malaysia; hafizi@aelb.gov.my; 3 Department of Electrical, Electronic & Systems Engineering, Faculty of Engineering and Built Environment, Universiti Kebangsaan Malaysia, Bangi 43600, Selangor, Malaysia; asma@ukm.edu.my (A.A.-S.); fadzilah.abdullah@ukm.edu.my (N.F.A.)

**Keywords:** radiation monitoring, remote monitoring, radiation sensor, radiation safety, Internet of Things, IoT, unmanned aerial vehicles, UAV, drone

## Abstract

As nuclear technology evolves, and continues to be used in various fields since its discovery less than a century ago, radiation safety has become a major concern to humans and the environment. Radiation monitoring plays a significant role in preventive radiological nuclear detection in nuclear facilities, hospitals, or in any activities associated with radioactive materials by acting as a tool to measure the risk of being exposed to radiation while reaping its benefit. Apart from in occupational settings, radiation monitoring is required in emergency responses to radiation incidents as well as outdoor radiation zones. Several radiation sensors have been developed, ranging from as simple as a Geiger-Muller counter to bulkier radiation systems such as the High Purity Germanium detector, with different functionality for use in different settings, but the inability to provide real-time data makes radiation monitoring activities less effective. The deployment of manned vehicles equipped with these radiation sensors reduces the scope of radiation monitoring operations significantly, but the safety of radiation monitoring operators is still compromised. Recently, the Internet of Things (IoT) technology has been introduced to the world and offered solutions to these limitations. This review elucidates a systematic understanding of the fundamental usage of the Internet of Drones for radiation monitoring purposes. The extension of essential functional blocks in IoT can be expanded across radiation monitoring industries, presenting several emerging research opportunities and challenges. This article offers a comprehensive review of the evolutionary application of IoT technology in nuclear and radiation monitoring. Finally, the security of the nuclear industry is discussed.

## 1. Introduction

Since the German physicist, Hans Wilhelm Geiger introduced the Geiger-Muller counter to measure radiation in July 1928, modern electrical devices have been adopted in radiation research [1]. With the advancement of science and technology, nuclear technology is being used in various fields, including energy production, healthcare, environmentalism, water, food and agriculture, astronomy, and other industries. While these fields benefitted from nuclear technology, the radiation emitted from nuclear materials poses risks to the associated personnel, the public, and the environment.

To negotiate the benefits of using nuclear technology while keeping humanity and nature safe, the Internal Commission on Radiological Protection (ICRP) states that “the likelihood of incurring exposures, the number of people exposed, as well as the magnitude of their individual doses, should be kept As Low As Reasonably Achievable (ALARA) taking into account economic and societal factors” [2]. This principle means the even with regard to a small dose, radiation should be avoided if receiving that dose does not provide a direct benefit. Nevertheless, if the work must be conducted, basic protective measures in radiation safety applying to time, distance, and shielding, must be adhered to so as to reduce the radiological risk. To achieve this, radiation detectors such as Geiger-Muller Counters, dose rate meters, personal dosimeters, and portal monitors are useful to provide information about the dose of radiation received, either directly or indirectly after the conversion of units has been performed, for precautionary and monitoring purposes.

The need for mobile radiation monitoring devices has increased with the increased usage of radiation technology for various purposes, such as regular monitoring at nuclear facilities and medical surveillance. Mobile monitoring is also crucial to ensure continuous monitoring in the occurrence of a radiological event such as the nuclear disaster of the Fukushima Daiichi Power Plant. As a result of this event, the radioactive substances are still being accumulated in the surrounding forest even six years post-accident. Despite challenging terrain and restricted access, the accumulated monitoring data were used for disaster mitigation plans by modelling and predicting the stochastic cancer risk [3]. In addition to the civil usage of radiation technology, radiation monitoring is important to curb the illicit transfer of radioactive materials and nuclear terrorism.

Not long after the September 11th tragedy, where the World Trade Center, New York, was hit by two aeroplanes and a third plane was crashed into the Pentagon, Washington DC, the International Atomic Energy Agency (IAEA) launched a three-year plan for enhanced anti-terrorism activities. The plan is known as the IAEA Nuclear Security Plan of Activities, aimed at reinforcing and strengthening nuclear security through a comprehensive and coordinated international approach [4]. While these radiation sensors help to monitor the level of radiation in certain designated areas and the level of radiation the radiation workers are exposed to, few attempts have been made to mount a different type of instrument in manned vehicles such as cars, aeroplanes, and helicopters [5], for a larger scale mission. Unfortunately, deploying manned vehicles in a nuclear accident site may expose the crew to a severe level of ionizing radiation [6].

A Wireless Sensor Network (WSN) was deployed to overcome the human safety issue by installing nodes to radiation sensors that can collect and transmit radiation data to a base station wirelessly [7]. Although this technology may assist in reducing the radiation exposure of radiation personnel, the packet transmission of large throughput from the scattered coverage of sensor nodes in the nuclear environment may lead to the failure of the central node, which can paralyze the entire radiation monitoring network. In 1989, the Internet of Things (IoT) emerged, as the TCP/IP protocol founder John Romkey introduced an automated toaster that can be turned on/off via the internet. It marks the beginning of Industrial Revolution 4.0, whereby devices are able to communicate with each other using the Internet. It can be seen as an upgraded version of WSN, through which each radiation sensor can be IP-enabled, and radiation data can be transmitted in real-time to the end-user.

Recently, unmanned aerial vehicles (UAVs), also known as drones, have become increasingly popular due to their mass production, and its cost-effectiveness. Consumers use UAVs for simple tasks such as photography and videography. Originally, the UAVs were intended to be used as an aerial torpedo in military operations, considered to be a flying bomb [8], but now, it offers an opportunity for use in many other fields. For example, drones are now used in cellular communication [9], emergency response [10], agriculture [11], wildlife monitoring [12], oil and gas [13], security and surveillance [14], mining [15], and medical delivery services [16]. Combining UAV technology and IoT technology in radiation monitoring makes the monitoring operation more efficient. As IoT technology becomes more pervasive and technology adoption occurs at a faster rate, various radiation sensors can easily be mounted on drones for desired tasks. It has grown to become a vital instrument in the nuclear industry. However, this opens a new set of challenges that will be discussed in this paper.

The contribution of this paper can be summarized as follows:

- Reviewing and establishing a classification of the available radiation sensors in the industry based on their technical functionalities and performances.
- The provision of a comprehensive review of the evolutionary application of IoT technology in nuclear and radiation monitoring, starts as early as the wireless sensor networks era until the current progress on the Internet of Things adoption.
- A further review of the potential use of Internet of Things technologies on Unmanned Autonomous Vehicles (UAV), specifically drones, in radiation monitoring. Furthermore, the current challenges presented by the current limitations in the radiation monitoring industry are summarized.

The rest of this paper is organized as follows: Section 2 will discuss the methodology of the review process. Section 3 is dedicated to Nuclear Radiation Sensors, wherein the classifications of radiation sensors are deliberated. Section 4 is focused on the application of these radiation sensors, and challenges to their deployment are mentioned in Section 5. Section 6 will review the Internet of Things and UAV application in radiation monitoring, while the challenges based on usage of IoT and drones in radiation monitoring will be described in Section 7. Finally, Section 8 concludes this paper.

## 2. Methodology

### 2.1. Eligibility Criteria

This review is more focused on the evolutionary technology used in radiation monitoring. Using the PICOS (Problem, Interception, Comparison, Outcome, Study Design) components [17], a variety of databases were explored, including peer-reviewed literature and the grey literature, for a general overview of the sensors.

- Problem: studies were selected with the focus on radiation monitoring sensors.
- Intervention: studies include the interventions before and after the introduction of the Internet of Things, with or without the inclusion of unmanned aerial vehicles.
- Comparison: studies were selected by comparing the methods used in performing radiation monitoring.
- Outcome: studies were considered as eligible if the radiation monitoring methods were in line with their evolutionary aspect.
- Study design: a mixture of experimental studies, books, proceedings, websites, articles from regulatory bodies as well as product specifications were considered for an overall review.

### 2.2. Search Strategy

Several databases were searched to obtain studies, including MDPI, IEEE, Elsevier, Springer and PubMed, among others. The search terms used were divided into four categories: detection, data transmission, data analysis and decision and drone-related, as shown in Table 1.

## 3. Nuclear Radiation Sensors

A sensor is a device or an element of a system used to measure physical, chemical, biological, and any other parameters, and is fundamental for monitoring, measurement, and control systems [18]. While radiation is defined as the energy emitted from a source through a medium in the form of waves and particles, from radio wave—the lowest wavelength, to gamma radiation—the highest energy in the electromagnetic spectrum.

**Figure 1:**
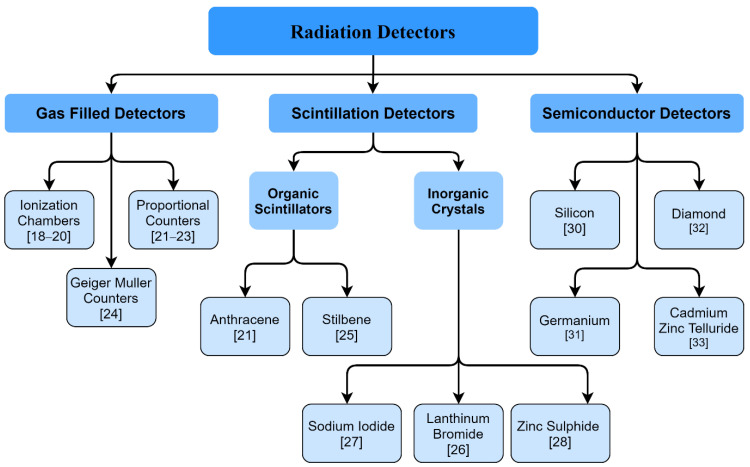
The classification of radiation detectors that are being used in radiation monitoring.

A nuclear detector is a unique instrument that is used to detect nuclear particles such as alpha particles, beta particles, gamma-ray, X-ray, proton, neutron, etc., based on the principle of ionization [19]. When a highly energetic nuclear particle enters a material medium, it ionizes the medium, and, through different sensor mechanisms, the radiation can be detected. The detection of such energy requires different types of sensors, broadly classified in Figure 1.

### 3.1. Gas-Filled Detectors

The gas-filled detector is the simplest detector, compared to a scintillator detector or a semiconductor detector. This type of detector consists of a metallic cylinder, or one made of other materials such as plastic, contains an electrode filled with inert gas as the medium and is connected to the pure capacitive load or an electrical circuit with a power supply, load resistor, and a signal. In the event that an incident ionizing radiation enters the medium; it ionizes the inert gas and produces ion-free electron pairs which are later subjected to the electric field. While the positive ion moves towards the surface of the cylinder, the free electron moves towards the electrode and into the electric circuit, generating an electric pulse or count before it returns to the metallic cylinder and recombines with the positive ion and becomes neutral again. There are three types of Gas-Filled Detectors—an ionization chamber, a proportional counter, and a Geiger-Muller Counter.

#### 3.1.1. Ionization Chamber

An Ionization Chamber is the simplest form of a gas filled detector that measures the exposure rate of X-ray and gamma radiation and the dose absorbed via the application of the Bragg-Gray principle, operating at a low voltage that does not induce an avalanche of electrons and does not have a dead time issue [20]. An ionization Chamber can detect alpha particles and beta particles with the application of thin window [21]. Applications that utilize ionization chambers include radiation survey instruments [22], a radiation source calibrator, and the remote sensing of ionization [23].

#### 3.1.2. Proportional Counter

The work in [24] states that the radial electric field between the cathode and the anode is generated by applying a positive high voltage to the anode, higher than the ionization chamber to induce the gas amplification phenomenon, which is the acceleration of free electrons from the initial ionization in a strong electric field inducing Townsend avalanche. This secondary type of ionization occurs at the threshold field of the order of 106 V/m [23]. The proportionality between the size of the output pulse and the total loss of energy as a result of the incident radiation defines the proportional counter, and is reliable for alpha and beta discrimination and used as a soft X-ray spectrometer for contamination screening [25], as well as for neutron detection [26].

#### 3.1.3. Geiger-Muller Detector

The Geiger-Muller (GM) tube follows the same mechanism as a proportional counter by inducing a Townsend avalanche, but the gas amplification caused by a single avalanche is higher, in the order of 106–108 [23]. The de-excitation of secondary free electrons releases an ultraviolet photon with sufficient energy to cause another avalanche originating from the gas or even the tube wall, and an uncontrolled chain avalanche occurs throughout the entire volume of gas in the cylinder. This entire process takes between 200 μs–400 μs to complete. During this time, the Geiger-Muller detector is considered to be dead, and incapable of detecting further nuclear particles. Adding alcohol, for example 10% ethanol, inside the gaseous tube may absorb the excess energy in the form of vibrational and rotational energy. Thus, it helps to reduce the dead time of the counter before it is able to count again. Since a low energetic particle can cause an avalanche across the entire chamber, the Geiger-Muller counter cannot differentiate the energy of the incident particles based on the pulse size for selective energy counting [26]. Nonetheless, the Geiger-Muller counter is a reliable instrument that can be used to detect the presence of charged particles, neutrons, and photons [27].

### 3.2. Scintillation Detector

Scintillation is the property of a material medium that when a charged particle enters, it absorbs its energy and leads to the emission of light. There are two types of scintillation, the first is counter—organic scintillation, such as anthracene [24] and stilbene [28] and the second is inorganic crystal scintillators such as lanthinum bromide (LaBr3) [29], sodium iodide (NaI) [30], and zinc sulfide (ZnS) [31]. The ultraviolet light formed in the scintillator focuses on a photocathode, thereby inducing a photoelectric effect, and later hits a series of dynodes with a different potential difference that will undergo amplification in the photomultiplier tube. The collection of the electrons is interpreted by a pulse amplifier. Besides gamma-rays and charged particles, a scintillation detector is often used in Wavelength Dispersive X-ray Fluorescent Spectrometer and can be applied to detect a high energy X-ray [32].

### 3.3. Semiconductor Detector

A semiconductor detector is an alternative to gas-filled and scintillation detectors. A compact detector, with a solid density of 1000 times greater than gas, can provide more carrier information for a given incident radiation event than is possible compared to other types of the detector [23]. When sing a PN junction diode doped with silicon [33], germanium [34], diamond [35], or cadmium zinc telluride (CZT) [36], the incident radiation enters the depletion region, causing the thermal excitation of the electron (~3 eV) from a valance band to a conduction band, creating electron-hole pairs in a reverse bias configuration. Through the collection electron-hole pairs, a detection signal is formed. Since the thermal excitation of an electron is low, a semiconductor detector can provide an enhanced energy resolution and is used for general charged particle spectroscopy [36], alpha particle spectroscopy [37], X-ray spectroscopy [36], gamma-ray [38], and personnel monitoring [39]. Unfortunately, this excellent detector is susceptible to performance degradation from radiation-induced damage.

## 4. Application of Radiation Detector for Monitoring Purposes

All celestial bodies, including the earth are products of certain energetic astrophysical processes known as nucleosynthesis, initiated by an explosive event called the big bang. After billions of years, and still ongoing, a series of nuclear processes, including fusion, neutron capture, proton capture, energetic particle interaction, and spallation, introduced the various nuclides known to us today [40]. This means that all living things are subjected to radiation exposure anywhere and at any time as radionuclides can be found naturally in air, soil, water, and food. According to [41], humans are exposed to radiation doses as high as 82% from cosmic and terrestrial sources, the inhalation of radioactive gas radon and its decay product inside any building, which all occur naturally. Furthermore, the ingestion of Potassium-40 in food can also lead to exposure [42,43]. Thus, radiation exposure needs to be measured whether the radiation originates from background radiation or any other nuclear activity, for safety purposes.

### 4.1. Personal Dosimetry

With these inevitable background radiation exposures all around us, regulatory bodies worldwide recommend a radiation dose limit to reduce the radiological risks to humans. Personal dosimetry can be used to monitor individual exposure. For general public exposure, the National Council on Radiation Protection and Measurement (NCRP) suggested the annual effective dose of 1 mSv for continuous exposure or 5 mSv if exposure is infrequent. Concurrently, the International Commission on Radiation Protection (ICRP) proposed an annual effective dose of 1 mSv or higher if needed as long as the average over 5 years does not exceed 1 mSv [44]. In Malaysia, the Atomic Energy Licencing Board (AELB) suggested the same annual effective dose of 1 mSv in a calendar year [45]. According to [40], the average annual effective dose from terrestrial radiations, including stones, trees, buildings, is around 0.28 mSv. This radiation dose is below the recommendations of both the NCRP and ICRP. Under normal circumstances, the general public is not required to track their level of exposure. In the Fukushima Daiichi Nuclear Power Plant accident, the residents who participated in the Fukushima Health Management Survey were provided with a glass badged personal dosimeter [46]. After 4 months of collecting data, the Fukushima Health Management Survey reported that as many as 66.3% of the 460,408 residents received the annual effective dose of less than 1 mSv, and 94.9% received less than 2 mSv [47]. Even though this has exceeded both the NCRP and ICRP recommendation, it is still considered safe because the exposure is below the occupational exposure.

For occupational exposure, the radiation personnel working in nuclear facilities are highly likely to be exposed to radiological hazards. The NCRP suggested an annual effective dose of 50 mSv, and the ICRP recommended 20 mSv, averaged over 5 years with a dosage of no more than 50 mSv in any one year [43] while the AELB suggested a 20 mSv annual effective dose in a calendar year [44]. Being exposed to radiation for too long can be biologically harmful. It can affect cells or lead to a change in DNA. This biological effect is categorized as a deterministic effect, a threshold of the dose received, over which biological damage and a stochastic effect is likely to occur, which is a probability that does not depend on the dose received [48]. Radiation monitoring using a personal dosimeter is compulsory for radiation personnel to manage this biological effect. Examples of personal dosimeters and their technical specifications are listed in Table 2.

On the other hand, a Thermo luminescence dosimeter (TLD) is a passive personal dosimeter used by more than 50% of radiation personnel worldwide in 2016 [49]. It is mainly used in medical physics and environmental monitoring [50]. Several types of TLDs such as calcium fluoride and lithium fluoride are placed in a TLD badge to detect low or high levels of gamma radiation, electron, and neutron energy, and need to be sent to a lab for analysis. Despite its high accuracy in detecting these ionization radiations, its main function is to monitor the accumulated dose received by the radiation personnel. However, if a radiological accident occurs and the instantaneous dose rate is high, TLD cannot provide dose rate readings or warn radiation personnel of such situations. To mitigate this limitation, [51] created an electronic personal dosimeter for mobile application by integrating a 5 mm^2^ CsI(Tl) detector, a 3 mm^2^ silicon photodiode detector, application-specific integrated circuit (ASIC), and a microcontroller unit (MCU). This system was built in a cylindrical shape and is connected to an android phone through an audio jack for power harvesting and data communication. A user interface window will display the dose rate in real-time, with three levels of radiation hazards, such as a normal radiation dose, threshold radiation dose, and intolerable radiation dose so that the radiation personnel can monitor their safety.

### 4.2. Environmental Monitoring

As mentioned before, the formation of the earth through a prolonged process of nucleosynthesis, resulted in a significant number of radionuclides. They can be found terrestrially and at sea, and can be categorized into: (i) primordial radionuclides, which includes the radionuclides that are not completely decayed and as old as the Earth; (ii) secondary radionuclides, the product of decayed primordial radionuclides, and (iii) cosmogenic radionuclides which are the product of stable nuclides being continuously bombarded by cosmic rays in the atmosphere [64]. The distribution of these radionuclides, with the addition of anthropogenic radionuclides from human activities, varies from one place to another, and the radioactivity should be monitored.

On 26 April 1986, the world was stunned by a nuclear event at the Chernobyl nuclear power plant. The major release of radioactive substances from Unit 4 lasted for ten days, resulting in an area of more than 200,000 km^2^ in Europe being contaminated with radioactive cesium [65]. The deposition was highly heterogenous, which was influenced by rain. As a result of the nuclear event, environmental monitoring was practised in many countries worldwide by establishing a remote station for monitoring purposes [66].

For the development of an intelligent environment monitoring system (SEM) that integrates air quality, water quality and radiation sensors, robust methods of machine learning, denoising methods, and the development of suitable wireless sensor networks are important. A high-pressure Xenon ionization chamber (HPXe) was introduced as a promising device for gamma-ray spectrometry to monitor environmental-based radiation. However, due to its high cost and unstable output reading, influenced by temperature variation, challenges exist in searching for stable and robust radiation sensors as compatible candidates for the SEM system [67].

Radioecological environmental contaminants are derived from multiple sources, ranging from source terms originating from power plants to the multiple contaminants from artificial radionuclides of industrial origins. The detection strategy must include benefits to both humans and the environment [68]. Therefore, new sensing devices utilizing IoT have been created, whereby data can be transferred wirelessly into a cloud system, and additional computing mechanisms can be accurately implemented for predicting the most significant radioactive contaminant in a specific area, or can collaboratively linked via multi-lateral diplomatic agreement.

Finally, Tritium radionuclide plays an important role in the environmental impact of radiology, especially in a Canada Deuterium Uranimum (CANDU) type of reactor. An analysis of tritiated water and gross alpha/beta currently utilizes liquid scintillation counter and gas proportional counter for air and water samples. Pathway assumptions, inhalation, ingestion, dose coefficients and laboratory measures have been identified as the primary source of uncertainties for dose estimation apart from the systematic minimum detection limits for many other environment samples [69]. Most importantly, the population living near the power plant must be protected from the environmental health impact through the provision of online and real-time tritium monitoring technology.

### 4.3. Different Monitoring Application

#### 4.3.1. Health

**Figure 2:**
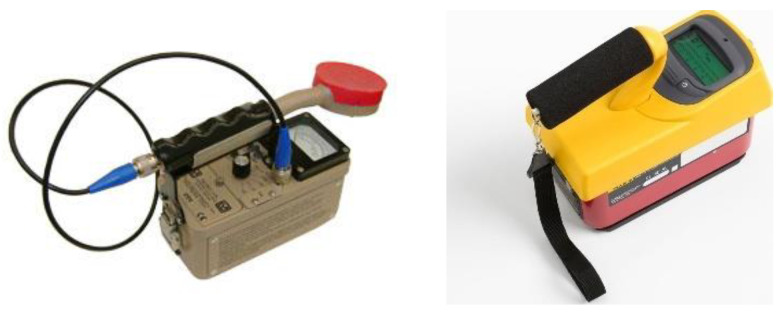
Examples of radiation survey meters used in radiation monitoring.

While it is true that radiation may cause biological damage to humans, the disruptive properties of ionizing radiation offer advantages in nuclear medicine. For decades, a low dose of X-ray has been used for nuclear imaging techniques, while a high radiation dose is used for cancer treatment. According to Chen and Kuo [70], a high dose of radiation therapy or radiotherapy itself has been used for at least two-thirds of cancer treatments across developed countries and is a vital curative treatment for uncomplicated locoregional tumours. In radioiodine therapy for thyroid cancer, patients are asked to ingest a small dose of 131 I, whereby the radioactive iodine will enter the bloodstream and accumulate at the thyroid gland, destroying the gland. For this treatment, patients have to be hospitalized for radiation monitoring, which can be performed using the ionization chamber survey meter, as shown in Figure 2. For example, in [71], the exposure rates are measured at a 5 cm distance from the stomach and neck and 1 m and 2 m from the patient sitting on the hospital bed. These readings are recorded every day until the patient is considered ready to be discharged. Apart from the thyroid gland, the ICRP has suggested the equivalent dose limit for other body parts, as stated in Table 3 below.

#### 4.3.2. Nuclear Reactor Facilities

According to the World Nuclear Association, 440 commercial nuclear power plants operate in 31 countries generating around 10% of the world’s electricity and 50 more reactors are under construction. Apart from his, around 220 research reactors used for research and training as well as for the production of medical and industrial isotopes are in operation in more than 50 countries [73]. Whether radioactive materials are being used to generate electricity, or for medical or industrial purposes, the methods through which the radioactive materials are to be used in the desired activity is complex. Their use requires much space in the nuclear vicinity, and these allotted areas are considered controlled radiation areas. As regulated by the AELB Regulation 2010 [74], nuclear facilities are one of the controlled radiation areas that the annual dose rate received by radiation personnel is expected to exceed three-tenths of the dose limit of 20 mSv in a calendar year. A continuous monitoring system consists of an alarm, and a precise readout at specific places should be installed. IAEA Safety Standards [75] suggested that external radiation monitoring systems should be installed in reactor containment rooms, that are adjacent to the refueling facilities of the containment area, spent fuel storage facility, fuel handling machine, treatment and storage facilities for radioactive waste, decontamination facilities, and as well as transport routes for fuel and waste.

### 4.4. National Security

**Figure 3:**
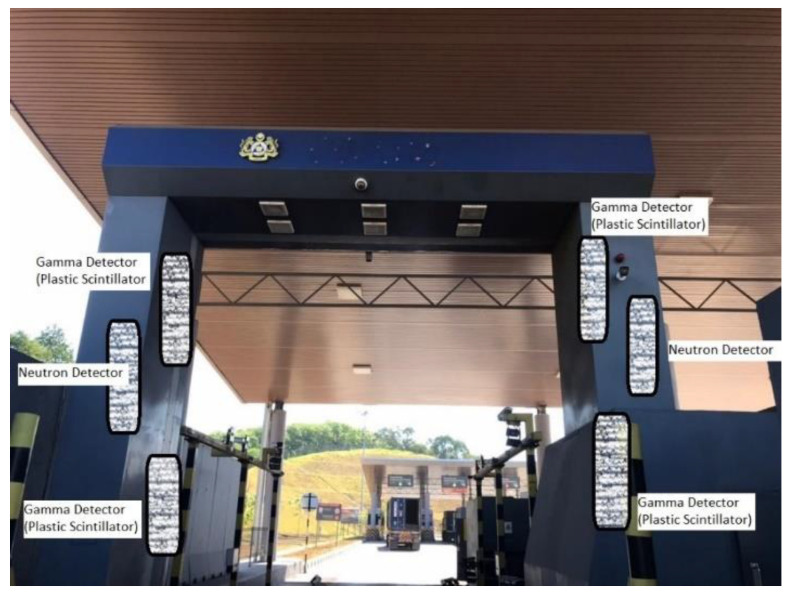
A radiation portal monitor at customs and immigration check point Bukit Kayu Hitam, Kedah, Malaysia.

Given the many benefits that result from nuclear technology, it is possible for nuclear technology to be used for other purposes. To avoid nuclear warfare, 191 states, including five nuclear-weapon states, have signed a Treaty on the Non-Proliferation of Nuclear Weapons, intending to prevent the spread of nuclear weapons and weapons technology while promoting the peaceful use of nuclear technology [76]. According to [77], container cargos, shipped in and out of seaports, transport 90% of world trade. Without strict inspections, seaports could act as an avenue for the illicit transfer or smuggling of radioactive materials such as uranium and plutonium, leading to terrorist attacks through the use of technology such as nuclear weapons and dirty bombs. An example of the Radiation Portal Monitors’ (RPMs) installation, a radiation detection system that is used to screen vehicles and cargo in and out of a country to improve national security, is shown in Figure 3. RPMs are assembled using a proportional counter with 3He gas, which is the gold standard for a thermal neutron detector in a moderating polyethylene box that detects neutrons with energies ranging from thermal to several MeV. Besides 3He, BF3 filled proportional detectors, boron-lined proportional detectors, scintillating glass fibre detectors, and scintillator coated wavelength-shifting fibre detectors can be used to detect slow and fast neutrons in RPM Kouzes 2010 [78].

An innocent alarm, produced via a radiation portal monitor when detecting a small amount of naturally occurring radiative material (NORM) contents in materials crossing the country or states, has always presented a challenge for front-line officers. Polyvinyl toluene (PVT)-based gamma-ray scintillation detectors have been identified as an optimum detection material. However, false alarms are still being detected; thus, spectral information alone is not able to distinguish between real and nuisance alarms. Artificial neural networks are now being used to discriminate NORM from other materials not under regulatory control (MORC) [79]. The Department of Homeland Security, USA has also re-examined the experimental data and computer simulations aimed at rapidly detecting localised radiation sources with a high detection rate probability for RPM facilities. PVT and NaI (Tl) detectors are involved in this test. The challenges fall within the scope of detecting unique nuclear material, including radiation dispersal device materials that may appear at borders [80].

## 5. Challenges in Radiation Monitoring

The three basic categorizations of radiation detectors for specific functions in radiation monitoring have helped to determine the source of radiation, whether it occurs naturally, results from human activities, or for potentially preventing countless possible attacks from nuclear adversaries. Despite the continuous advancement of detection and monitoring radiation technology, limitations still exist, which can be addressed and improved on in the subsections below.

### 5.1. Safety of the Radiation Monitoring Worker

Traditionally, radiation monitoring is performed manually, where radiation personnel carry radiation detectors to locate radiation sources or radioactive materials. While this offers exceptional spatial resolution, radiation personnel are subjected to radiation exposure. The ICRP proposed a guide for radiation safety in which the exposure should be kept as low as reasonably achievable by reducing the time of exposure, increasing the distance from the source to reduce the exposure according to inverse square law, and through the use of a shielding material such as lead gown while monitoring the radiation. As safety is the main priority in the nuclear industry in general, a technology that can monitor radiation remotely without compromising the efficiency and accuracy of radiation data will eliminate the ICRP concern.

### 5.2. Time-Consuming Radiation Data Gathering

One of the issues of performing backpack monitoring other than safety reasons is that the process of radiation monitoring is time-consuming. Given that the size of nuclear facilities or even the area of a radioactive fallout, resulting from a nuclear event, that needs monitoring is huge, performing backpack monitoring seems impractical. A radiation detector can be installed on a system that uses a vehicle to perform radiation monitoring, and this can be achieved with the help of unmanned ground vehicles (UGVs) such as cars, vans, helicopters and unmanned aerial vehicles (UAVs) or drones. Desirable high payload radiation detectors can be mounted on these vehicles, improving the efficiency and accuracy of radiation monitoring, and reducing the manual monitoring time significantly.

### 5.3. Topological Challenging Environments

In some cases, monitoring needs to be performed in hostile, inaccessible areas of an explosion, decommissioned buildings or other topologically challenging environments such as jungles, where manned or unmanned vehicles are unable to access. A smaller UAV or drone can be deployed to perform the same mission.

### 5.4. Real-Time Decision Making

Some countries, including France, Japan and South Korea have a high number and high density of nuclear power plants per km^2^ [81]. The risk associated with their operation needs to be constantly evaluated. In extreme cases such as a nuclear accident, real-time radiation monitoring and data analyses are critical for decision-making. However, some systems rely heavily on preset criteria for decision-making and not on real-time data analytics profiting from multiple sensor deployment [82,83]. In the case of the Fukushima Power Plant accident, a 30 years old monitoring system, SPEEDI was blamed for the disaster due to the failure to release information to help evacuation [84]. This can be counteracted through the introduction of the Internet of Things (IoT), where smart radiation sensors can communicate with end-users with the help of the Internet, which will provide real-time data. Most IoT systems can also profit from the analytics system, whereby different data analysis tools or procedures can be applied to the generated IoT devices’ data. Valuable information can then be derived to provide decision support, which is helpful to initiate decontamination, evacuation processes, or any other necessary decisions.

## 6. Internet of Things in Radiation Monitoring

The study in [85] showed that radon gas causes approximately 21,000 deaths annually from lung cancer and it is the second contributing factor of lung cancer after smoking. According to Dr. Michael Repacholi, the World Health Organization’s Radiation and Environmental Health Unit coordinator, radon is the main source of exposure to ionizing radiation, and accounts for 50% of the public’s exposure to naturally-occurring radiation sources in many countries [86]. Hence, continuous radiation monitoring is essential for a location to protect its inhabitants from serious health hazards. It is also important to analyze the impact of radiation on the environment [58].

Thanks to the advancement in wireless communication and electronics, sensors have become smart, small in dimension, lower in cost and power consumption, and multi-functional and can communicate within a short distance [87]. In this regard, WSN technology, as a vital enabler for the IoT, is certainly useful in radiation monitoring, in which a large number of sensor nodes can be connected to monitor radiation across a wide area. Although WSN technology is promising, it has its limitations. According to [88], the drawbacks of WSN technology are its limited resources, limited processing capability, memory, and by the fact that, to provide the power required for an improved sensing resolution in a noisy environment, sensor nodes need to be deployed in a higher density. This is where the Internet and cloud overcome the limitation.

**Figure 4:**
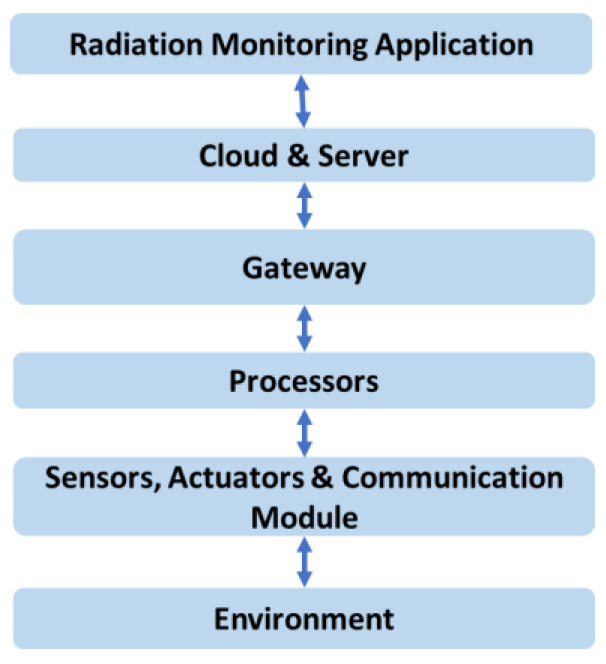
General layers of Internet of Things in radiation monitoring.

With recent developments in IoT and the growth of networking within industrial machines, the deployment of IoT has become a new revolutionary method through which to develop the radiation monitoring industry. Therefore, in recent years, many academic and industrial units have developed surveillance tools and techniques to monitor radiation issues at a much faster rate than traditional tools and methods. By definition, IoT is the network of all things, including non-living objects and living things that can be identifiable, embedded with intelligence sensing capabilities, and able to exchange data over the Internet [89]. The IoT ecosystem consists of web-enabled smart devices that use embedded systems such as processors, sensors and communication hardware to collect, send and act on data acquired from their environments. IoT devices share the physical data collected from the sensors to an IoT gateway or to other edge devices, where data is either sent to a cloud or analyzed locally to form the application layer. In other words, the Cyber-Physical System (CPS) in IoT involves sensing, computing, controlling, and communication between physical components (e.g., smart sensors, devices, systems, and human beings) and cyber components (e.g., cloud and big data centres). Therefore, the communication system module plays an important role in interfacing the CPS. In IoT, the main competing communication technologies are Wi-Fi, Bluetooth, ZigBee, Near Field Communication (NFC), 2G/3G/4G/5G cellular, as well as Low Power Wide Area Network (LPWAN), which includes Long Range (LoRa), SigFox and Narrowband IoT (NB-IoT). A detailed comparison of these different technologies for smart environmental monitoring can be found in [67,90]. The general diagram of IoT for radiation monitoring is shown in Figure 4.

The emergence of IoT has not occurred spontaneously. It has been driven by many factors, such as the convergence of operating and information technology, analytics at the edge, virtualization and cloud, a technology explosion, digital transformation, and enhanced user interface [89]. All of these factors together improve the efficiency, accuracy of devices and increase the economic benefits. Pursuing these aims made the IoT a reality and categorized them into several verticals—transportations, retail, industrial, energy, oil and gas, finance, agriculture, farming, and nuclear industry, especially for radiation monitoring. In the context of IoT radiation monitoring, the focus is usually placed on assessment and recovery, such as the coordination of emergency responses. The following subsections elaborate on existing IoT-based radiation monitoring, which can be categorized into two groups: stationary and mobile radiation monitoring, using UAV systems.

### 6.1. Stationary Radiation Monitoring

Brennan et al., [91] introduced a radiation detection system with a wireless sensor network (WSN) in 2004. The nodes are equipped with static monitoring devices such as Area Gamma Monitors (AGM) for data procurement. In another WSN setting, another monitoring system was developed using fixed radiation sensors that were installed throughout a nuclear plant and using a mobile Personal Digital Assistant (PDA) to form a Mobile Ad-Hoc Wireless Network (MANET) [92]. WSN-based radiation monitoring was also set up, using sensor nodes connected to AGMs and router nodes to establish a wireless multi-hop communication to route data to a central monitoring station [93].

The long-term recording of data for radiation monitoring is cost-intensive. Using a smart sensor system to process data before the output is recorded decreases the level of data transmitted and the computing power that is required [94]. While the use of smart sensor systems reduces operational cost, it also provides a seamless data transfer for remote monitoring that aids decision making. The authors of [95] investigated the potential of IoT to realize an integration platform based on cost-effective WSN for radiation monitoring. The authors proposed a system architecture for radiation monitoring based on an open-source IoT platform, called ThingsBoard, consisting of Message Queue Telemetry Transport (MQTT) messaging strategy, and LoRaWAN protocol. It was revealed that the new WSN-IoT platform provides numerous advantages over the conventional WSNs, such as more network flexibility and scalability, minimal hardware and software requirements, cloud data management, and big data processing. In [96], a Geiger-Muller counter is interfaced with a firmware called NodeMCU to update measurement and detection of radiation to a radiation IoT platform also using MQTT as the means of communication. The Geiger-Muller counter is also equipped with a Global Positioning System (GPS) module to obtain the radiation location, and this data can be monitored in real-time through a web server.

The same approach was studied in [97] by proposing a prototype named RadMon. This prototype is a system that is used for radiation and meteorological monitoring and consists of radiation and meteorological devices for data producers, early warning and decision support systems for data consumers and provides data visualization that utilizes MQTT for sending field measurement data to a centralized storage system. It also includes a representational state transfer (REST) web service for the interaction between storage systems with web-based user interfaces and open-source databases. Several data parameters, collected by RadMon, including the radiation dose rate, solar radiation, power supply, wind speed, air pressure and temperature, are displayed on a web-based dashboard.

The study in [98] modified a commercial radon gas sensor embedded with System-on-Chip that provided additional intelligence and communication and was placed in buildings such as homes and research labs. The system is able to monitor the radiation value and project it to the user through a web dashboard. In a smart home setting, this system can activate mitigation mechanisms such as forced ventilation to reduce the concentration of radon gas if there is an accumulation of radon. The authors in [99] developed an IoT-based monitoring system prototype to remotely monitor the air quality and ionizing radiation level of the environment. The system consists of: (i) sensing nodes to measure environmental parameters. Each sensor node has been built with an ESP8266 and a WiFi-enabled Arduino compatible microcontroller whereby the data was transferred to a remote server based on the MQTT protocol; (ii) A central server for data processing, data storage, and real-time analysis; (iii) A nearly real-time web and Android application that can be operated via remote computers and mobile phones to provide remote access.

The authors of [100] proposed a modest wide territory sensors arrangement to detect the radiation leakage around atomic reactors. The system was designed based on an open-source server, Things Speak Web, and a set of cost-effective sensors to monitor atmospheric parameters such as temperature, smoke, humidity, sound, and carbon monoxide. A cost-effective IoT-based system was developed in [101] for radiation monitoring which was comprised of a Geiger counter, Wemos microcontroller, and a temperature, humidity, and light ambient sensor. The collected data can be stored and observed in real-time via the web interface. In addition, the measurement results showed that there is no correlation between temperature/humidity and radiation in the considered area in Chittagong, Bangladesh, but there is a correlation between radiation and light intensity.

The authors in [102] developed a radiation monitoring device to support the radiological emergency preparedness system in the Yogyakarta Nuclear Area, Indonesia. The device was designed as a sensor network to acquire radiation data from the environment to be stored in the database server. The developed system consists of a Geiger-Muller detector, high voltage power supply, signal conditioning system, and Arduino as the counter and data processor. The collected data can be transmitted to a server through a wireless network using the node MCU communication module. The data can then be used to analyze the nuclear emergence potential in the nuclear emergency response and preparedness system.

For the communication of radiation detection, if exclusively active nodes can be selected, the network lifetime can be optimized, as can resource allocation, and the reliability of collected data. The study in [103] is the only work conducted on radiation monitoring as of yet to address the problem of active node selection for radiation localization on IoT networks. The proposed method is based on data-driven active node selection that dynamically reads data from currently active nodes to select future nodes, considers the achieved coverage area by the sensors, and considers parameters such as residual energy, power cost, and data confidence levels in the selection process.

In terms of wireless transmission, [104] Studied the ability of LoRA (Long Range), a Low Power Wide Area Networks (LPWAN) to transmit data wirelessly over long-range for nuclear radiation monitoring. The radiation dose measured by the Gamma radiation sensor was transmitted wirelessly over a secured and reliable connection over 10 km in rural and 7 km in urban areas. Most recently, the team from the European Organization for Nuclear Research (CERN) developed Waste Radiation Monitoring (W-MON), an end-to-end data infrastructure for thousands of highly sensitive and ultralow-power gamma sensors for environmental radiation monitoring based on LoRA [105]. The system proposed a web-based user application for real-time monitoring, data visualization and status control for all devices based on open-source tools to improve their integration into the overall CERN Radiation and Monitoring Unified Supervision service. The firmware was designed to minimize power consumption, using confirmed uplink and downlink messages strategy activation.

Compared to manual operation, the IoT-based radiation monitoring system can enhance operators’ safety, speed up monitoring procedures, improve the data collection efficiency, improve the chances of secure communication, and reduce operational costs. Although static radiation monitoring strategies provide numerous advantages over manual data collection, it still faces some technical limitations, such as the time and costs required to deploy WSN infrastructure in the considered area. This problem becomes more acute when the considered area is contaminated with radioactive contaminants.

### 6.2. Mobile Radiation Monitoring Using UAV

#### 6.2.1. Radiation Monitoring Using Conventional UAV

Although radiation monitoring can be performed on foot using handheld or backpack equipment that provides an excellent spatial resolution, the area of nuclear facilities is huge, so such a survey seems unrealistic. The deployment of unmanned vehicles reduces the time of manual monitoring significantly. The advantage of an unmanned survey over a manned survey is its low flight altitude and narrower line spacing, resulting in more effective monitoring, especially in hot spot activities. Moreover, an unmanned survey system can be used in areas that are hazardous to humans. By integrating smart radiation sensors with microprocessor and wireless communication devices and mounting them on any UAV, radiation monitoring in nuclear power plants or any associated activities can be performed remotely with minimal exposure for the radiation worker. Compared to the traditional method of radiation monitoring, flying a drone reduces the operation time significantly, and improves all of the issues of nuclear technologies such as safety, security, and safeguarding.

Since the Fukushima Daiichi accident, many researchers have been interested in drone technology for radiation monitoring. According to Miroslav Pinak, Head of the IAEA Radiation Safety and Monitoring Section, “UAV-based technologies will be crucial for advancing radiation and improving long-term monitoring of contaminated areas” [106]. The IAEA has been working with the Fukushima Prefecture in 2012 in developing and applying drones for radiological monitoring. From 2012 to 2020, the IAEA has assisted Fukushima Prefecture in providing a complete UAV-based instrumentation system for radiation measurements and post-measurement analysis and interpretation methodology under the IAEA Action Plan on Nuclear Safety framework.

The work in [107] quantitatively and qualitatively analyzes UAV-based radiation sensor systems. The authors examined various UAVs, radiation sensors, and radiological survey missions and categorized them by mission. In addition, they proposed a new figure of merit (FOM) formula that explains the mutual effects of parameters of both radiation sensors and UAVs on system performance. The proposed FOM can be used to efficiently assess whether the system achieves the required minimum detectable activity (MDA) without field tests. Based on the identified constraints from the FOM and MDA score, the authors provided several nuclear plant accident scenarios. It was shown through the MDA score that although a larger radiation sensor enhances photoelectron efficiency, it negatively increases the mass effects of UAV endurance when fast flight speed and high flight altitudes are preferred for wide-range monitoring.

In [108], research on radiation contamination mapping was performed using GPS waypoint and mounting a CZT detector on a drone. The drone received the data at the altitude of 2.5 m which was validated by comparing it with a ground survey at the height of 1 m. A similar approach was taken in [109] with different altitudes between 1–10 m height, for which the obtained data is presented in a colour scale heat map. Furthermore, [110] developed a radiation detection and mapping prototype for theoretical nuclear disaster response. A Teviso RD3024 radiation sensor, based on an array of customized PIN, was used to measure Cs-137 and Co-60 using a search algorithm to identify a safe path for a human to travel.

In [111], the researcher integrated a Compton gamma-ray detector with an optical camera on a drone hovering at 1.5 m from a cesium source to visualize the radiation distribution. A stick PC on the drone took 10 min to reconstruct the image before transference to the base PC via Wi-Fi. While the technique adopts the IoT technology, hovering for an extended time for image reconstruction may limit the operation of the drone in one flight.

The authors in [112] developed a remote radiation imaging system comprising a lightweight Compton camera and a 3D LiDAR mounted on a multi-copter drone to remotely measure the distribution of radioactive substances. The Compton camera mounted on the drone has the ability to visualize a 3D distribution of radioactive substances in difficult-to-return zones. The system was tested in the Hama-dori region, Fukushima, Japan, where the drone realized 3D visualization of several hotspots. In [113], a drone equipped with CsI(Tl) and SiPM was used to blindly locate a Cs-137 source in one of three boxes via a comparison of the radiation spectra with background radiation. A similar method was used in [114] to locate a lost source. The experiment was performed using an NaI(Tl) scintillator detector. A I-131 was placed at three different locations before the drone was located using the source location algorithm based on the inverse square law.

For a usability experiment, [115] uses a CZT detector on an octocopter drone in a coaxial configuration with the aim to localize the nuclear radiation source, for which the location is unknown to the drone operators, and can only be configured with the help of a 3DOF haptic device and a 3D augmented reality screen displayed on a computer screen. The evaluation was carried out via the allocation of the NASA-TLX questionnaire and the SPAM (situation present assessment method) to 10 drone operators and evaluating it mental demand, performance, effort, and frustration or stress.

In [116], a drone was used to detect radioactive material and classify the target’s radioactivity in transit. The authors proposed a motion planning framework by integrating visual and inertial localization approaches, in which a navigation function was constructed based on the available knowledge regarding the 3D workspace and the drone dynamics. The navigation function is able to avoid obstacles and generate a safe path to the moving target. The performance of the proposed approach was tested in a simulation environment. However, the framework’s performance needs to be examined in the presence of sensor noise and odometry errors.

The authors in [117] developed and examined a mini-drone gamma-ray spectrometer. The gamma-ray spectrometer has two 103 cm3 BGO scintillation detectors mounted on a hexacopter. The field measurements were obtained at a low flight altitude (5 m to 40 m) and a low flight speed of 1 m/s. The results revealed that the gamma-ray field rapidly decreases with an increasing flight altitude, which highlights the significant impact of flight altitude. It was shown that the flight altitude for mini-airborne surveys could be up to 40 m and may take into account all important conditions including the size and intensity of an assumed anomaly, detector sensitivity, flight speed, and vegetation characteristics. The developed drone is able to detect size-limited radiation anomalies with a comparable quality to a standard airborne survey.

In [118], an advanced gamma radiation detector was developed for UAV operation to exploit drones’ flight and payload capability at under 25 kg. To measure the gamma energy spectra and determine the direction of radioactivity, eight CsI(Tl) crystals were used, which utilised silicon photomultipliers. A small-sized drone with a 6 kg lift capability, and with up to 40 min of endurance, was used for development and measurement. The performance of the developed system was examined in both laboratory and outdoor trials. The results present how the developed system’s directional responses can be used to indicate the source location in real-time and to guide UAV during the survey mission.

The authors in [119] designed an IoT-based device to determine the absorbed dose of gamma and UV radiation. A set of sensors, including a humidity and temperature meter, UV grove radiation meter, Geiger-Muller meter, and height and atmospheric pressure meter were connected to a particle electron microcontroller. Then, the collected data were transferred to a Raspberry Pi for further processing, storage, and transmission to the cloud. The radiation-measuring prototype was mounted on a hexacopter drone. The developed system was compared with meters calibrated in certified laboratories, where the validation results matched those obtained by the other devices, with an error of 2%.

The authors of [120] equipped a quadcopter small-sized drone with a sensitive gamma detector to coordinate the flight based on the measured gamma data and to detect the small dose radiation distribution in a given area. One of the limiting factors faced when attempting to increase flight height is the sensor sensitivity, as a higher sensitivity would measure the highest possible altitude. In [121] a UAV path planning system was proposed for radiation dose mapping for a meter-level resolution. Two algorithms were used for path planning, including: (1) a flood-fill algorithm to plan a path within each chunk of adjacent void areas, and (2) a 2-opt algorithm for path planning between the chunks of void areas. In an affected area near the Fukushima Daiichi Nuclear Power Plant disaster, the field measurement showed that the proposed method is able to successfully minimize the overall flight time compared to the results obtained from the 2-opt only and flood-fill algorithms.

To enhance the safety of personnel during the cleanup process of nuclear facilities, the work in [122] developed a semi-autonomous UAV-based radiation cleanup system that is able to sense radiation contamination remotely, obtain sample contaminations of low-energy byproducts, and perform cleanups. In addition, the authors addressed the issue of quadrotors exerting all the required forces in all six DoF. The developed multirotor drone can exert arbitrary forces and torques, independently and instantaneously, and this improvement can allow UAVs to respond to external disturbances quickly and to maintain their position with precision during the mission.

After a severe accident at the Chernobyl Nuclear Power Plant in 1986, many liquidation materials that had been contaminated by the radioactive fallout were buried in so-called Radioactive Waste Temporary Storage Places (RWTSPs). Until 2020, more than 700 burial sites have been thoroughly investigated, but the location of around 300 burial sites remains unknown. The authors of [123] used sensor technologies such as UAV-based LiDar and multi-spectral imagery and combined the prominent features generated from the high-resolution sensors with a random forest classifier to detect the location of TWTSPs in the Chornobyl exclusion zone.

The authors of [124] used a UAV-based survey system to detect unknown radioactive biomass deposits in Chernobyl’s exclusion zone. UAV-based LiDar data, multi-spectral, and gamma spectrometry data, along with the machine learning methods, were used to precisely map trenches and clamps. Two different UAVs were used for measurements; the LiDar measurements were taken via an octocopter with a flight time of up to 20 min, while multi-spectral measurements were taken via Quantum Trinity VTOL with a flight time of up to 50 min. The measurement rate of the octocopter was 18.5 kHz, the flight altitude was 50 m, and the flight speed was 4–7 m/s. In comparison, the VTOL flight altitude and flight speed were 130 m and 17 m/s, respectively. The measurement results have shown that integrating UAV-based LiDar, multi-spectral image technology, and aerial gamma spectrometry surveys can successfully produce a map for the considered zone, with an overall accuracy of 95.6–99.0%.

UAV-based monitoring systems are considered to be a promising solution for radiation monitoring purposes, whereby the sensors are able to collect data which they analyze onboard, or send to the cloud for storage, instead analyzing the data remotely. These devices can also remotely identify the potential issues within an area and provide notify operators and even perform automated interventions without human interactions. This allows for faster response time, less risk of exposure, lower overall operational cost, and more reliable information compared to static monitoring strategies. However, while UAV-based monitoring systems provide new opportunities they create some critical technical challenges, which will be discussed in detail in the next section.

#### 6.2.2. Radiation Monitoring Using IoT and Internet of Drones

By integrating smart radiation sensors with microprocessor and wireless communication devices and mounting them on a drone, radiation monitoring in nuclear power plants or any associated activities can be performed remotely with minimal exposure to the radiation worker. In [111], the researcher integrated a Compton gamma-ray detector with an optical camera on a drone, hovering at 1.5 m from a cesium source to visualize radiation distribution. A stick PC on the drone reconstructed the image in 10 min before transferring it to the base PC via Wi-Fi. While this technique adopts IoT technology, hovering for an extended time for image reconstruction may limit the operational ability of the drone in one flight.

The authors in [119] designed an IoT-based device to determine the absorbed dose of gamma and UV radiation. A set of sensors such as a humidity and temperature meter, UV grove radiation meter, Geiger-Muller meter, and height and atmospheric pressure meter were connected to a particle electron microcontroller. Then, the collected data was transferred to a Raspberry Pi for further processing, storage, and transmission to the cloud. The radiation-measuring prototype was mounted on a hexacopter drone. The developed system was compared with meters calibrated in certified laboratories, where the validation results matched those obtained by the other listed devices, with an error of 2%.

In addition, many studies and machine learning algorithms have been developed for autonomous drone systems, in which a drone can determine and accomplish its mission even in the absence of remote control from a Ground Control Station (GCS). This requires the development of autonomous path finding that avoids obstacles from buildings and terrains. The authors of [124] used a UAV-based survey system to detect unknown radioactive biomass deposits in Chernobyl’s exclusion zone. UAV-based LiDar data, multi-spectral, and gamma spectrometry data, along with the machine learning methods, were used to precisely map trenches and clamps. Two different UAVs were used for measurements; the LiDar measurements were obtained via an octocopter with a flight time of up to 20 min, while multi-spectral measurements were obtained via Quantum Trinity VTOL with a flight time of up to 50 min. The measurement rate of the octocopter was 18.5 kHz, the flight altitude was 50 m, and the flight speed was 4–7 m/s. In comparison, the VTOL flight altitude and flight speed were 130 m and 17 m/s, respectively. The measurement results have shown that integrating UAV-based LiDar, multi-spectral image technology, and aerial gamma spectrometry surveys can provide a successful map of the considered zone with an overall accuracy of 95.6–99.0%.

The most recent UAV network architecture that has been developed is a group of operatively interacting drones, known as the Internet of Drones (IoD). A multi-fleet of drones for nuclear power plant (NPP) monitoring, consisting of main drone fleets and a reserve drone fleet structure has been proposed in [125]. The multi-fleet of drones provides additional communication subsystems, IoD and private cloud-based data processing to support a crisis center decision-making system. The IoD’s multi-connection self-organizing network mesh topology and the use of cloud services provide various links between drone and cloud, telemetry and payload data access over the internet, real-time access to drone control, and at the same time ensures secure communication through encryption and authentication mechanisms. The radio frequency vulnerabilities of IoD have been highlighted in [126]. Furthermore, [127] presented an intelligent and secure fog-aided IoD that can jointly optimize energy consumption while fulfilling the quality of service (QoS) requirements. Other UAV networks security considerations have also been proposed in [126].

UAV-based monitoring systems are considered to be a prominent solution for radiation monitoring purposes, whereby the sensors can collect data and either analyze the data onboard or send it to the cloud for storage, after which it is analyzed remotely. These devices also can remotely identify the potential issues within an area and provide a notification/alarm to operators and even perform automated interventions without human interactions. This allows for a faster response time, less risk of exposure, lower overall operational cost, and more reliable information compared to static monitoring strategies. Although UAV-based monitoring systems pose new opportunities they also create some critical technical challenges, which will be discussed in detail in the next section.

## 7. Challenges and Opportunities

**Figure 5:**
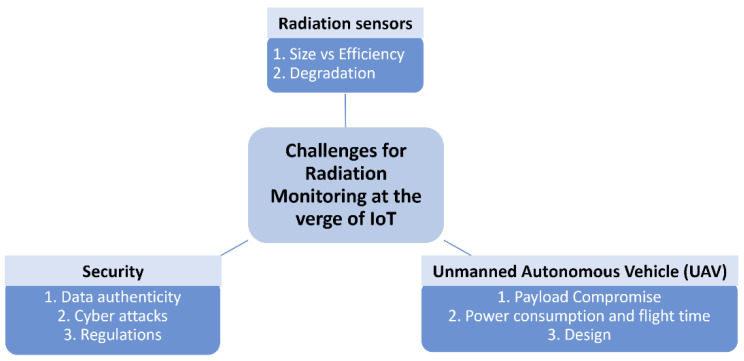
Summary of the challenges of radiation monitoring at the verge of the Internet of Things.

The IoT dramatically improved the radiation monitoring industry by introducing increased efficiency, accuracy, decision support, safety and time-saving. However, as is the case for any other new emerging technology, the application of IoT in radiation monitoring presents a new set of challenges that need to be reviewed. The subsection below classifies these challenges into three categories—radiation sensors, drones, and security. Figure 5 summarizes the challenges of radiation monitoring at the verge of the Internet of things.

### 7.1. Radiation Sensors

Radiation sensors are the heart of radiation monitoring, whereby radiation data is collected, and several issues have been raise that can be improved on.

#### 7.1.1. Size vs. Efficiency

Many researchers in radiation monitoring have used CZT detectors due to their high-Z material, which is suitable for room temperature gamma radiation detection [128] and provides high energy resolution. Nevertheless, the sensitivity of the detector is dependent on its dimension [129]. Due to its limited volume of 1 mm^3^, the counting efficiency is relatively low compared to other radiation detectors [109]. Using a larger dimension of CZT can yield better efficiency.

#### 7.1.2. Degradation

Measuring the radiation fluency emitted from radioactive sources, especially in harsh conditions, is critical for a long period. Although radiation data can be received continuously, the radiation sensors are being subjected to degradation. According to Diggins [130], a variation in sensor output exists if the radiation monitoring system’s sensing element or auxiliary electronics are significantly impacted by radiation. A diagnostic of the radiation sensor is required to measure the degradation before the system reaches the total failure point. The authors in [49,50,51,98,131,132] conducted a radiation hardness experiment on electrical components by exposing them to a high level of radiation. They found that an exposure of 100 Gy for more than 5 h does not degrade the electrical components. However, the 2D range scanner malfunctioned after being exposed for an additional 2 h and 49 min, with a total dosage of 124 Gy received. Meanwhile, the charge-coupled device (CCD) camera degraded and produced a blueish image after its exposure for an additional 3 h and 51 min with the total dose received of 169 Gy. The radiation exposure may be even higher in a nuclear accident, therefore, further research should be conducted regarding the radiation hardness of sensors and electrical devices.

### 7.2. Unmanned Autonomous Vehicle (UAV) Technology

Cost-effectiveness and ease of maneuvering are benefits that have been discussed, making UAVs, especially drones, a popular choice as radiation monitoring vehicles. However, these devices have their own limitations that can be overcome so as to better assist the monitoring system.

#### 7.2.1. Payload Compromise

The payload is defined as the added weight that is independent of the weight of the drone itself. The performance of radiation monitoring is not as simple as deploying a vehicle with radiation sensors. In reality, in order to achieve a better sensor output, other external factors need to be considered. The temperature of the ignition radioactive materials, the radioactive plume or the temperature of the gaseous particles releases to the atmosphere, and vision can be helpful tools in long-distance surveillance. These complementary factors require separate sensors to improve an understanding of the situation, but compromises the drone’s power consumption. To overcome this issue, carbon fiber reinforced plastic can be used as opposed to other common materials such as fibreglass, aramid fibre, thermosets and thermoplastics [133] to reduce the weight of the drone frame due to its low density, although it is more resilient than steel. In this way, more sensors can be mounted, and with less weight, this drone can safely travel in nuclear facilities and access confined areas.

#### 7.2.2. Power Consumption and Flight Time

One of the main limiting factors of mini-airborne surveillance systems is their short operational time. Most UAVs are equipped with a lithium battery that can be operated for a short duration of time, of around 20 min, depending on the payload of the vehicles. For a small monitoring area, the aim might be feasible, but for large spatial monitoring, the lithium battery may not be sufficient to monitor radiation in a single operation flight.

An improved power supply option is required for UAV endurance and to reduce operational costs. Electrochemical reactions, producing electricity using hydrogen fuel cells to combine with oxygen from the air, can be used to replace traditional lithium batteries. The research in [134], using 500 g of hydrogen cell fuel, investigated a propulsion system on UAV with an average propulsion power of 314 W and managed to power the flight for 26 h at a specific energy of 1170 Wh/kg. Compared with a lithium battery of approximately 200 Wh/kg, the hydrogen fuel cell offers six times more capacity than the lithium battery.

Alternatively, solar power panels can be installed for longer flight times as opposed to lithium batteries. In [135], a test was conducted, with a UAV weighing less than 4 kg, that achieve a nine-hour minimum flight time. As optimistic as this seems, the location, the date, as well as solar irradiation should be taken into account, as the experiment was performed during the summer in Cranfield, UK, and the flight time may reduce during other seasons or in other parts of the world.

The problem of energy limitation can be partially addressed by improving localization and path planning strategies. Conventionally, UAVs perform their mission based on a set of waypoints or by using offline optimization methods to optimize the mission path. However, the performance of such methods is not optimized and results in inefficient flight, i.e., a longer time is needed to scan an area, especially in a dynamic environment where the environmental situation is constantly changings. In this regard, path planning optimization, autonomous navigation, and localization of radioactivity are increasing topics of interest in environmental monitoring.

Similarly, UAV-based monitoring systems that can analyze data either on board, on the edge or send by sending data to the cloud for remote analysis also consume energy [136]. Data submission to the cloud or the use of a bigger drone can reduce energy consumption for data processing, but the computation offloading, and wireless data transmission will both be sacrificed. Energy management strategies for mobile devices from hardware to software aspects, especially on drones are the focus of many studies, such as [137], which proposes a system architecture for inter-connected drones to allow for the usage of deep learning on edge and on the cloud. The authors in [138,139] propose other architectures based on blockchain technology to demonstrate the prolonged drone operating time, but conclude that efficiency and reduced consumption are still not sufficient for rescue and search operations. More optimized architectures and transmission strategies are desired to make the paradigm shift in this aspect.

Another limiting factor for increasing the efficiency of a drone missions is the poor performance of sensors at high flight speeds. In this regard, AI-based digital signal processing and machine learning techniques can enhance the performance of detection algorithms at higher flight speeds.

#### 7.2.3. Design

The design of the UAV systems also performs an important role in radiation monitoring as it directly impacts power consumption in terms of flight time and the reliability of radiation monitoring and mapping. Each type of drone has its advantages and disadvantages in relation to radiation monitoring. An appropriate drone selection depends on the user’s aims. Multirotor drones are not suitable for long-distance monitoring due to their limited endurance as most of the energy is used to combat gravity, while fixed-wing drones necessitate skill training and require a runway or a catapult launcher to set their course and a parachute or a net for landing. Nevertheless, even after acknowledging its limitation, a drone remains a valuable tool to measure radiation. A technique was recently developed by Belgium’s Nuclear Research Center and the Belgian aeronautical firm, Sabca, that uses fixed-wing drones that hover autonomously for an extended period and a multirotor that can carry heavier payloads [140].

Aside from the types of drones, the effect of aerodynamic turbulence from rotary wings may influence the response of the radiation sensors for mapping radiation plumes [125,141]. Since the direction of radiation sensors is important, the placement of radiation sensors on drones should be determined to avoid the collection of inaccurate data. A computational fluid dynamics simulator (CFD) or a numerical fluid dynamics simulation can be used to identify the best placement for a radiation sensor on the drone, while providing quantity readings of lift and drag and field properties such as pressure and velocities. According to [141], the radiation sensor should be placed at the centre of the drone body as the radioactive particles are always concentrated at the highest volume in such a position.

### 7.3. Security

Initially, the focus of IoT development was on the consumer sector and has latterly attracted the attention of other industries. In this regard, IoT devices were first created for the commercial retail market and were not assessed to ensure that the data was safe, which raised security concerns. Security is the biggest concern in the nuclear industry, since the term nuclear itself is always a public concern, and is critical for sustaining nuclear activities [142]. For example, some of the prototypes were developed, ensuring either little or no protection of information, resulting in data being completely accessible. Therefore, each system needs to be assessed in term of reliability, safety, and accuracy before the implementation of such IoT systems as any data related to radiation is extremely sensitive and should be protected.

#### 7.3.1. Data Authenticity

Performing radiation monitoring in real-time, whereby all the radiation data is sent to the Internet or cloud may streamline operations, but the radiation data sent from radiation detectors are vulnerable and can be stolen or altered by an unauthorized person. The leaking of this information or the spread of tempered data may result in serious consequences, including panicking the public. Introducing blockchain technology to IoT that provides public and private keys to information can prevent this data from being manipulated [143]. Depending on the sensitivity of the radiation data, Public Permissionless Blockchain, Public Permissioned Blockchain, Private Permissionless Blockchain, or Private Permissioned Blockchain may be used to control who can access the radiation data.

#### 7.3.2. Cyber Attacks

Data alteration is not the only concern arising from communication loopholes; hackers will also exploit this opportunity. An analysis of drone system security has been performed by this author [144], and finds that UAVs are prone to spoofing where information can be captured, modified, or injected and also prone to malware infection, which allows hackers to create a reverse-shell TCP payload to install malware on the running systems and the ground stations. Besides this risk, UAVs are also vulnerable to interception and jamming that can compromise radiation monitoring work and lead to the loss of UAV autonomy. This security attack can be managed by monitoring the incoming and outgoing network traffic to detect anomalies by incorporating Intrusion Detection Systems in the UAV systems.

#### 7.3.3. Regulations

All of the reviewed studies have one aim, which is to look for ways to improve the radiation monitoring industry. However with the general proliferation of drones, threats may be encountered as a result of the vulnerability of such systems. Taking the case of a man who landed a 50 cm drone carrying a small camera and a plastic bottle containing unidentified contents with a mark of a symbol that warns of radioactive material on the rooftop of the Japan Prime Minister’s office [145], security issues related to drones leads to safety concerns regarding the potential danger of drones. Similarly, given the case of an employee of the United States government intelligence agency, who lost control of a DJI Phantom drone that had evaded radar detection and crashed at the White House [146], regulations on commercial drone technology need to be examined.

A review paper in [147] stated that approximately half of all countries do not have regulations regarding the use of UAVs for civil applications. In order to improve security, UAV regulations should focus on targeting the regulated use of airspace by UAVs, imposing operational limitations, and tackling the administrative procedures of flight permissions, pilot licenses and data collection authorization.

After the Japanese Prime Minister’s rooftop office incident, The Act on Prohibition of Flying UAVs over Important Facilities and Their Peripheries was promulgated with the establishment of a restricted no-fly area inside a 300-m radius of important facilities, including nuclear facilities. Similarly in Malaysia, all drone activity must follow the Civil Aviation Regulation 2016 (MCAR) Regulation 140–144 [148].

## 8. Conclusions

Radiation detection has been a major factor in the nuclear industry. The introduction of the Internet of Things (IoT) created a paradigm shift in radiation monitoring by maximizing the protection of the public or of personnel from radiation exposure and preventing countless potential radiation threats using advanced data analyses and prediction approaches. This paper reviewed the evolutionary application of IoT technology in nuclear and radiation monitoring, where data can be remotely stored, analyzed, and used for decision making or for taking any action. The technical functionality and performance of radiation sensors have been reviewed and established. The potential use of IoT in radiation monitoring and the challenges based on current limitations in the radiation monitoring industry have been explored. By integrating sensors with unmanned vehicles, radiation data can be gathered safely and accurately. By observing the trend of IoT, it can be posited that more smart radiation sensors will be made in the future with better efficiency and accuracy, that are smaller in size and weight and have a lower power consumption to allow the radiation monitoring industry to become further automated, whereby faster and more timely decision making can occur, for the purpose of human protection.

## Figures and Tables

**Table 1:** The search term used in this review.

Categories	Search Term
Detection-related	Radiation Detector Gas-Filled Detector Scintillator Detector Semiconductor Detector
Data transmission-related	Wireless Sensor Network Internet of Things IoT
Data analysis and decision-related	Radiation monitoring data analysis
Drone-related	Unmanned Aerial Vehicles Unmanned Aerial System UAV & Drone

**Table 2:** Technical specification based on the commercial availability of radiation personal dosimeter.

**Specifications**	Canberra Industries Inc. UltraRadiac-Plus [52]	Far West Technology Inc. Canary IV [53]	Fisher Scientific Inc. RadEyeTM PRD-ER [54,55]	Fuji Electric Corporation of America DOSE-i [56]	Polimaster Inc. PM1621MA [57]	Polimaster Inc. PM1703MO-1BT [58]	Polimaster Inc. PM1704A-M [59]	RAE Systems by Honeywell GammaRAE II R [60]	X-Z Lab. Inc. RadPavise [61]	Mirion Tchnologies RADOS DIS-1 [62]	Mirion technologies DMC 3000TM [63]
**Detector Used**	Geiger Muller	PIN Diode	Sodium iodide dopen thalum NaI(Tl)	Silicon (Si) semiconductor	Geiger Muller	Geiger Muller, Cesium iodide doped thalium (CsI(Tl))	Geiger Muller, CsI	CsI, PIN Diode	Si photomultiplier, Yttrium orthosilicate (YSO) scintillator	Ionization chamber, Direct Ion Storage	Si chip
**Measurements**	Dose rate: 1.0 μR/h to 200 R/h (0.01 μSv/h–2 Sv/h) Cumulative Dose: 0.1 μR–999 R (0.001 μSv–999 Sv)	Exposure: 0.01–9999.99 mR/h (0.1 mGy–99,999.9 mGy)	Dose equivalent: 1 μrem/h–10 rem/h (0.01 μSv/h–100 mSv/h)	Dose equivalent Accumulated Dose: 0.001 mSv–999.9 mSv (0.1 mrem–99.99 mrem) Dose Rate: 0.001 mSv/h–999.9 mSv/h (0.1 mrem/h–99.99 mrem/h) ≤±10% (0.01 mSv–999.9 mSv (1 mrem–99.99 rem), 137 Cs)	Dose equivalent: 0.01 μSv–9.99 Sv ±10% (in range 1 μSv–9.99 Sv (100 μR–999 R)) dose equivalent rate: 0.1 μSv/h–1.00 Sv/h ±(10 + 0.0015 Ḣ + 0.01 Ḣ)%	Dose equivalent: 0.1 µSv–9.99 Sv dose equivalent rate: from 0.1 µSv/h–9.99 Sv/h	Up to 1300 R/h	Dose equivalent rate: 1 µR/h–600 R/h (0.01 µSv/h–6 Sv/h ± 20%	Dose rate: 1 μrem/h–100 rem/h (0.01 μSv/h–10 Sv/h)	Dose Equivalent: 1 µSv– 1 Sv	Dose Equivalent: 0.01 µSv–100 Sv
**Calibration**	N/A	1 year	2–3 years	1 year	N/A	N/A	Routine use—6 months	Routine—2 years Storage—3 years	1 year	6 months	9 months
**Trigger** **Parameter**	Flash, Audible, vibration	Audible	Audible	Audible	Visual, audible, vibration	Audible, vibration, light	Audible, visual	Audible, vibration, light	Audible, vibration, visual	N/A	Visual, audible, tactile
**Functional Temperature**	–22 °F to 141 °F (–30 °C to 61 °C) at up to 93% humidity.	Up to 50 °C	−4 °F–122 °F at up to 90% humidity	−10 °C to 40 °C (14 °F to 104 °F) at up to 90% humidity	−40 °F–140 °F at up to 98% humidity	−30 to +50 °C at up to 98% humidity	−4 °F–122 °F at up to 98% humidity	4 °F–122 °F at up to 95% humidity	−4 °F–122 °F at up to 90% humidity	−10 °C–50 °C at up to 90% humidity	−10 °C–50 °C at up to 90% humidity

**Table 3:** The equivalent dose limit for specific body parts was proposed by the ICRP [72].

Body Part	Equivalent Dose Limit
The lens of the eye	150 mSv
Skin (1 cm^2^ of highly irradiated)	500 mSv
Hands and feet	500 mSv

## Data Availability

Not applicable.

## References

[1] Shampo M.A., Kyle R.A., Steensma D.P. (2011). Hans Geiger—German Physicist and the Geiger Counter. Mayo Clin. Proc..

[2] IRCP (2008). 2007 Recommendations of the International Commission on Radiological Protection. Ann. ICRP.

[3] Mochizuki S., Kataoka J., Tagawa L., Iwamoto Y., Okochi H., Katsumi N., Kinno S., Arimoto M., Maruhashi T., Fujieda K. (2017). First demonstration of aerial gamma-ray imaging using drone for prompt radiation survey in Fukushima. J. Instrum..

[4] El Baradei M. Nuclear terrorism: Identifying and combating the risks. Proceedings of the International Conference on Nuclear Security: Global Directions for the Future.

[5] Pöllänen R., Toivonen H., Peräjärvi K., Karhunen T., Ilander T., Lehtinen J., Rintala K., Katajainen T., Niemelä J., Juusela M. (2009). Radiation surveillance using an unmanned aerial vehicle. Appl. Radiat. Isot..

[6] Kurvinen K., Smolander P., Pöllänen R., Kuukankorpi S., Kettunen M., Lyytinen J. (2005). Design of a radiation surveillance unit for an unmanned aerial vehicle. J. Environ. Radioact..

[7] Gomaa R.I. (2020). Performance analysis of wireless sensor networks for nuclear medicine applications. J. Radiat. Res. Appl. Sci..

[8] Sahm V.H., Werrell K.P. (1987). The Evolution of the Cruise Missile. Technol. Cult..

[9] Zulkifley M., Behjati M., Nordin R., Zakaria M. (2021). Mobile Network Performance and Technical Feasibility of LTE-Powered Unmanned Aerial Vehicle. Sensors.

[10] Zwęgliński T. (2020). The Use of Drones in Disaster Aerial Needs Reconnaissance and Damage Assessment—Three-Dimensional Modeling and Orthophoto Map Study. Sustainability.

[11] Behjati M., Noh A.M., Alobaidy H., Zulkifley M., Nordin R., Abdullah N. (2021). LoRa Communications as an Enabler for Internet of Drones towards Large-Scale Livestock Monitoring in Rural Farms. Sensors.

[12] Hodgson J.C., Baylis S., Mott R., Herrod A., Clarke R.H. (2016). Precision wildlife monitoring using unmanned aerial vehicles. Sci. Rep..

[13] Mattar R.A., Kalai R. (2018). Development of a Wall-Sticking Drone for Non-Destructive Ultrasonic and Corrosion Testing. Drones.

[14] Hell P.M., Varga P.J. (2019). Drone Systems for Factory Security and Surveillance. Interdiscip. Descr. Complex Syst..

[15] Kirsch M., Lorenz S., Zimmermann R., Tusa L., Möckel R., Hödl P., Booysen R., Khodadadzadeh M., Gloaguen R. (2018). Integration of Terrestrial and Drone-Borne Hyperspectral and Photogrammetric Sensing Methods for Exploration Mapping and Mining Monitoring. Remote Sens..

[16] Zailani M.A.H., Sabudin R.Z.A., Rahman R.A., Saiboon I.M. (2020). Drone for medical products transportation in maternal healthcare: A systematic review and framework for future research. Medicine.

[17] Amir-Behghadami M., Janati A. (2020). Population, Intervention, Comparison, Outcomes and Study (PICOS) design as a framework to formulate eligibility criteria in systematic reviews. Emerg. Med. J..

[18] Mukhopadhyay S.C., Mason A. (2013). Smart Sensors, Measurement and Instrumentation.

[19] Kandan V., Hassan M.F., Omar N., Shahar H., Mohamad F., Karim M.K.A., Sani S.A., Bradley D., Noor N.M. (2021). Advanced glow curve analysis of fabricated fibres for various sources of ionizing radiation. Radiat. Phys. Chem..

[20] Jamil M.Z.A.M. (2019). Effect of gamma irradiation on magnetic gadolinium oxide nanoparticles coated with chitosan (GdNPs-Cs) as contrast agent in magnetic resonance imaging. Radiat. Phys. Chem..

[21] Mettler F.A., Guiberteau M.J. (2012). Essentials of Nuclear Medicine Imaging: Expert Consult-Online and Print.

[22] Kaur A., Sharma S., Mittal B. (2012). Comparison of sensitivity of Geiger Muller counter and ionization chamber based survey meters. J. Nucl. Med..

[23] Knoll G.F. (2010). Radiation Detection and Measurement.

[24] Owaki S., Kimura Y., Kawanishi M. (1970). Scintillation Pulse Shapes of Anthracene Single Crystals in Nanosecond Region. II. Difference between Scintillation and Fluorescence Pulses. J. Phys. Soc. Jpn..

[25] Tuo X., Mu K., Li Z., Li X. (2008). Tritium Monitor Based on Gas-flow Proportional Counter. J. Nucl. Sci. Technol..

[26] Braby A., Badhwar G.D. (2001). Proportional counter as neutron detector. Radiat. Meas..

[27] Habrman P. (2019). Directional Geiger-Müller detector with improved response to gamma radiation. J. Instrum..

[28] Weldon R. (2020). Characterization of stilbene’s scintillation anisotropy for recoil protons between 0.56 and 10 MeV. Nucl. Instrum. Methods Phys. Res. Sect. Accel. Spectrometers Detect. Assoc. Equip..

[29] Mouhti I., Elanique A., Messous M.Y. (2018). Validation of a NaI (Tl) and LaBr3 (Ce) detector’s models via measurements and Monte Carlo simulations. J. Radiat. Res. Appl. Sci..

[30] Akkurt I., Gunoglu K., Arda S. (2014). Detection efficiency of NaI (Tl) detector in 511–1332 keV energy range. Sci. Technol. Nucl. Install..

[31] Ji J., Colosimo A.M., Anwand W., Boatner L.A., Wagner A., Stepanov P.S., Trinh T.T., Liedke M.O., Krause-Rehberg R., Cowan T.E. (2016). ZnO Luminescence and scintillation studied via photoexcitation, X-ray excitation and gamma-induced positron spectroscopy. Sci. Rep..

[32] Nakayama K., Nakamura T. (2013). X-Ray Fluorescence Spectroscopy for Geochemistry. Treatise on Geochemistry.

[33] Casse G. (2020). New trends in silicon detector technology. J. Instrum..

[34] Alexiev D., Reinhard M.I., Mo L., Rosenfeld A.R., Smith M.L. (2002). Review of Ge detectors for gamma spectroscopy. Australas. Phys. Eng. Sci. Med..

[35] Liu L., Ouyang X., Zhang J., Zhang X., Zhong Y. (2014). Polycrystalline CVD diamond detector: Fast response and high sensitivity with large area. AIP Adv..

[36] Cherry S.R., Sorenson J.A., Phelps M.E. (2012). Physics in Nuclear Medicine e-Book.

[37] Ruddy F., Seidel J., Chen H., Dulloo A., Ryu S.-H. (2006). High-resolution alpha-particle spectrometry using 4H silicon carbide semiconductor detectors. IEEE Trans. Nucl. Sci..

[38] Suslick K.S. (2001). Encyclopedia of physical science and technology. Sonoluminescence and Sonochemistry Massachusetts.

[39] Terasaki K., Fujibuchi T., Murazaki H., Kuramoto T., Umezu Y., Ishigaki Y., Matsumoto Y. (2017). Evaluation of basic characteristics of a semiconductor detector for personal radiation dose monitoring. Radiol. Phys. Technol..

[40] Marshall C.P., Fairbridge R.W. (1999). Encyclopedia of Geochemistry.

[41] Shahbazi-Gahrouei D., Setayandeh S., Gholami M. (2013). A review on natural background radiation. Adv. Biomed. Res..

[42] Ramachandran T. (2011). Background radiation, people and the environment. Int. J. Radiat. Res..

[43] Haghparast M., Ardekani M.A., Navaser M., Refahi S., Najafzadeh M., Ghaffari H., Masoumbeigi M. (2020). Assessment of background radiation levels in the southeast of Iran. Med. J. Islam. Repub. Iran.

[44] NCRP (1992). Limitation of Exposure to Ionizing Radiation.

[45] Atomic Energy Licensing Act 1984. https://www.aelb.gov.my/malay/dokumen/perundangan/RADIATION%20PROTECTION%20LICENSING%20REGULATIONS%201986.pdf.

[46] Miyazaki M. (2017). Using and explaining individual dosimetry data: Case study of four municipalities in Fukushima. Asia Pac. J. Public Health.

[47] Kamiya K., Ishikawa T., Yasumura S., Sakai A., Ohira T., Takahashi H., Ohtsuru A., Suzuki S., Hosoya M., Maeda M. (2016). External and Internal Exposure to Fukushima Residents. Radiat. Prot. Dosim..

[48] Choudhary S. (2018). Deterministic and Stochastic Effects of Radiation. Cancer Ther. Oncol. Int. J..

[49] Pradhan A.S., Lee J.I., Kim J.L. (2016). On the scenario of passive dosimeters in personnel monitoring: Relevance to diagnostic radiology and fluoroscopy-based interventional cardiology. J. Med Phys..

[50] Azorin J. (2014). Preparation methods of thermoluminescent materials for dosimetric applications: An overview. Appl. Radiat. Isot..

[51] Sholom S., McKeever S.W.S. (2015). Integrated Circuits from Mobile Phones as Possible Emergency Osl/Tl Dosimeters. Radiat. Prot. Dosim..

[52] UltraRadiacTM Plus. https://mirion.s3.amazonaws.com/cms4_mirion/files/pdf/spec-sheets/c0556_urad_spec_sheet.pdf?1562600527.

[53] Canary IV Dosimeter, Model 4084. https://www.fwt.com/hpi/hpi_4084ds.htm.

[54] RadEye™ PRD/PRD-ER Personal Radiation Detector. https://www.thermofisher.com/order/catalog/product/4250671#/4250671.

[55] Goldhagen P. (2012). Private Correspondence.

[56] Electronic Personal Dosimeter Dose-i. https://www.fujielectric.com/products/radiation/personal/dosei.html.

[57] X-Ray and Gamma Radiation Personal Dosimeter PM1621MA. https://en.polimaster.com/catalog/personal-dosimeters/x-ray-and-gamma-radiation-personal-dosimeter-pm1621ma/#tab-2.

[58] Personal Combined Radiation Detector/Dosimeter PM1703MO-1BT. https://en.polimaster.com/catalog/prd-gamma/personal-combined-radiation-detector-dosimeter-pm1703mo-1-bt/#tab-2.

[59] PM1704 Series. https://www.cbrnetechindex.com/Print/6331/polimaster-inc/pm1704-series.

[60] GammaRAE II R. https://safety.honeywell.com/content/dam/his-sandbox/products/gas-and-flame-detection/documents/Manual_GammaRAE-II-R_047-4505-000_RevD.pdf.

[61] RadPavise Personal Radiation Detector. https://www.x-zlab.com/product/radpavise-personal-radiation-detector/.

[62] DIS-1 Dosimeter. https://dosimetry.web.cern.ch/sites/dosimetry.web.cern.ch/files/download/DIS-1_UserManual.pdf.

[63] DMC 3000TM Personal Electronic Dosimeter. https://mirion.s3.amazonaws.com/cms4_mirion/files/pdf/spec-sheets/151199en-j_dmc_3000.pdf?1607366119.

[64] National Research Council (1999). Evaluation of Guidelines for Exposures to Technologically Enhanced Naturally Occurring Radioactive Materials.

[65] Anspaugh L. (2008). Environmental consequences of the Chernobyl accident and their remediation: 20 years of experience. Chernobyl.

[66] Assafiri Y., Nasreddine M., Roumie M. (1998). Early warning environmental radiation monitoring system. Environ. Sci..

[67] Ullo S.L., Sinha G.R. (2020). Advances in Smart Environment Monitoring Systems Using IoT and Sensors. Sensors.

[68] Brit S. (2009). Challenges in radioecology. J. Environ. Radioact..

[69] Hamlat S., Thompson P., Rinker M., St-Amant N., Pan P., Peters K., Dagher E., Jovanović S., Sauvé K. (2018). Independent environmental monitoring and public dose assessment around the Canadian Nuclear Power Plants. J. Radioanal. Nucl. Chem..

[70] Chen H.H.W., Kuo M.T. (2017). Improving radiotherapy in cancer treatment: Promises and challenges. Oncotarget.

[71] Ravichandran R., Al Saadi A., Al Balushi N. (2014). Radioactive Body Burden Measurements in 131Iodine Therapy for Differentiated Thyroid Cancer: Effect of Recombinant Thyroid Stimulating Hormone in Whole Body 131Iodine Clearance. World J. Nucl. Med..

[72] Kai M., Homma T., Lochard J., Schneider T., LeComte J., Nisbet A., Shinkarev S., Averin V., Lazo T. (2020). ICRP Publication 146: Radiological Protection of People and the Environment in the Event of a Large Nuclear Accident. Ann. ICRP.

[73] Nuclear Power in the World Today. https://www.world-nuclear.org/information-library/current-and-future-generation/nuclear-power-in-the-world-today.aspx.

[74] Regulation 8 Atomic Energy Licensing (Basic Safety Radiation Protection) Regulations 2010. https://radia.moh.gov.my/project/new/radia/FileTransfer/downloads/files/10BSS-2010_BI.pdf.

[75] Iaea Safety Standards Series No. NS-G-1.13. https://www-pub.iaea.org/MTCD/Publications/PDF/Pub1233_web.pdf.

[76] Treaty on the Non-Proliferation of Nuclear Weapons (NPT). https://www.un.org/disarmament/wmd/nuclear/npt/text.

[77] Pellens V. Transport of NORM in the port of Antwerp: From megaports to a special purpose measurement methodology. Proceedings of the 6th International Symposium on Naturally Occurring Radioactive Material.

[78] Kouzes R.T., Ely J.H., Erikson L.E. (2010). Neutron detection alternatives to 3He for national security applications. Nucl. Instrum. Methods Phys. Res. Sect. Accel. Spectrometers Detect. Assoc. Equip..

[79] Kangas L.J., Keller P.E., Siciliano E.R., Kouzes R.T., Ely J.H. (2008). The Use of Artificial Neural Networks in PVT-Based Radiation Portal Monitors. Nucl. Instrum. Methods Phys. Res. A.

[80] Siciliano E.R., Ely J.H., Kouzes R.T., Milbrath B.D., Schweppe J.E., Stromswold D.C. (2005). Comparison of PVT and NaI(Tl) Scintillators for Vehicle Portal Monitor Applications. Nucl. Instrum. Methods Phys. Res. A.

[81] Number of Operable Nuclear Reactors Worldwide as of May 2021, by Country. https://www.statista.com/statistics/267158/number-of-nuclear-reactors-in-operation-by-country/.

[82] Urso L., Astrup P., Helle K., Raskob W., Rojas-Palma C., Kaiser J. (2012). Improving evaluation criteria for monitoring networks of weak radioactive plumes after nuclear emergencies. Environ. Model. Softw..

[83] Hofman R., Pecha P., Šmídl V. (2014). Evaluation of detection abilities of monitoring networks using multiple assessment criteria. Int. J. Environ. Pollut..

[84] Funabashi Y., Kitazawa K. (2012). Fukushima in review: A complex disaster, a disastrous response. Bull. At. Sci..

[85] Vogeltanz-Holm N., Schwartz G.G. (2018). Radon and lung cancer: What does the public really know?. J. Environ. Radioact..

[86] Health Risk of Radon. https://www.epa.gov/radon/health-risk-radon#who.

[87] Tiwari P., Saxena V.P. (2015). Wireless sensor networks: Introduction, advantages, applications and research challenges. HCTL Open Int. J. Technol. Innov. Res..

[88] Lin R., Wang Z., Sun Y. Wireless sensor networks solutions for real time monitoring of nuclear power plant. Proceedings of the Fifth World Congress on Intelligent Control and Automation (IEEE Cat. No. 04EX788).

[89] Rayes A., Salam S. (2017). Internet of Things from Hype to Reality.

[90] Elmustafa S.A., Mujtaba E.Y. (2019). Internet of things in smart environment: Concept, applications, challenges, and future directions. World Sci. News.

[91] Brennan S., Mielke A., Torney D., MacCabe A. (2004). Radiation detection with distributed sensor networks. Computer.

[92] Barbarán J., Diaz M., Esteve I. (2007). RadMote: A mobile framework for radiation monitoring in nuclear power plants. Int. J. Electron. Circuit. Syst..

[93] Ebenezer J., Murty S.S. Deployment of Wireless Sensor Network for radiation monitoring. Proceedings of the 2015 International Conference on Computing and Network Communications.

[94] Lin T.-H., Liaw D.-C. (2014). Development of an intelligent disaster information-integrated platform for radiation monitoring. Nat. Hazards.

[95] Moriello R.S.L., Tocchi A. (2020). Exploiting IoT-Oriented Technologies for Measurement Networks of Environmental Radiation. IEEE Instrum. Meas. Mag..

[96] Muniraj M., Qureshi A.R., Vijayakumar D., Viswanathan A.R., Bharathi N. Geo tagged internet of things (IoT) device for radiation monitoring. Proceedings of the 2017 International Conference on Advances in Computing, Communications and Informatics.

[97] Susila I.P., Kusuma G., Isnaini I. (2018). Development of IoT based meteorological and environmental gamma radiation monitoring system. AIP Conf. Proc..

[98] Blanco-Novoa O., Fernández-Caramés T.M., Fraga-Lamas P., Castedo L. (2018). A Cost-Effective IoT System for Monitoring Indoor Radon Gas Concentration. Sensors.

[99] Tambasafidy F.P.E., Ratongasoandrazana J.B. (2019). IoT-based Environmental and Ionizing Radiation Monitoring System. Int. J. Innov. Res. Sci. Eng. Technol..

[100] Ashwini S.R., Harish B.R., Karthik R., Bafna K.D. Wireless Sensors Network for environmental radiation monitoring using IOT. Proceedings of the 2018 3rd IEEE International Conference on Recent Trends in Electronics, Information & Communication Technology.

[101] Mahatab T.A., Muradi M.H., Ahmed S., Kafi A. Design and analysis of IoT based ionizing radiation monitoring system. Proceedings of the 2018 International Conference on Innovations in Science, Engineering and Technology.

[102] Abimanyu A., Akmalia R., Salam M. (2020). Design of IoT-based Radiation Monitor Area for Nuclear and Radiological Emergency Preparedness System in Yogyakarta Nuclear Area. J. Phys. Conf. Ser..

[103] Alagha A., Singh S., Mizouni R., Ouali A., Otrok H. (2019). Data-Driven Dynamic Active Node Selection for Event Localization in IoT Applications—A Case Study of Radiation Localization. IEEE Access.

[104] Goyal J., Khandelwal A. Long range nuclear radiation monitoring system using LPWAN technology. Proceedings of the 2020 IEEE Sensors Applications Symposium (SAS).

[105] Manzano L.G., Boukabache H., Danzeca S., Heracleous S.D.N., Murtas F., Perrin D., Pirc V., Alfaro A.R., Zimmaro A., Silari M. (2021). An IoT LoRaWAN Network for Environmental Radiation Monitoring. IEEE Trans. Instrum. Meas..

[106] New Drone Technology for Radiological Monitoring in Emergency Situations. https://www.iaea.org/newscenter/news/now-available-new-drone-technology-for-radiological-monitoring-in-emergency-situations.

[107] Lee C., Kim H.R. (2019). Optimizing UAV-based radiation sensor systems for aerial surveys. J. Environ. Radioact..

[108] MacFarlane J., Payton O., Keatley A., Scott G., Pullin H., Crane R., Smilion M., Popescu I., Curlea V., Scott T. (2014). Lightweight aerial vehicles for monitoring, assessment and mapping of radiation anomalies. J. Environ. Radioact..

[109] Martin P.G., Moore J., Fardoulis J.S., Payton O.D., Scott T.B. (2016). Radiological Assessment on Interest Areas on the Sellafield Nuclear Site via Unmanned Aerial Vehicle. Remote Sens..

[110] Cai C., Carter B., Srivastava M., Tsung J., Vahedi-Faridi J., Wiley C. Designing a radiation sensing UAV system. Proceedings of the 2016 IEEE Systems and Information Engineering Design Symposium (SIEDS).

[111] Sato Y., Ozawa S., Terasaka Y., Kaburagi M., Tanifuji Y., Kawabata K., Miyamura H.N., Izumi R., Suzuki T., Torii T. (2017). Remote radiation imaging system using a compact gamma-ray imager mounted on a multicopter drone. J. Nucl. Sci. Technol..

[112] Sato Y., Ozawa S., Terasaka Y., Minemoto K., Tamura S., Shingu K., Nemoto M., Torii T. (2020). Remote detection of radioactive hotspot using a Compton camera mounted on a moving multi-copter drone above a contaminated area in Fukushima. J. Nucl. Sci. Technol..

[113] Baeza J., Valencia D., Baeza A. Use of drones for remote management of the close measure of radioactivity sources. Proceedings of the IGARSS 2018—2018 IEEE International Geoscience and Remote Sensing Symposium.

[114] Gong P., Tang X.-B., Huang X., Wang P., Wen L.-S., Zhu X.-X., Zhou C. (2019). Locating lost radioactive sources using a UAV radiation monitoring system. Appl. Radiat. Isot..

[115] Aleotti J., Micconi G., Caselli S., Benassi G., Zambelli N., Bettelli M., Zappettini A. (2017). Detection of Nuclear Sources by UAV Teleoperation Using a Visuo-Haptic Augmented Reality Interface. Sensors.

[116] Yadav I. (2018). Visual-Inertial Target Tracking and Motion Planning for UAV-based Radiation Detection. arXiv.

[117] Šálek O., Matolin M., Gryc L. (2018). Mapping of radiation anomalies using UAV mini-airborne gamma-ray spectrometry. J. Environ. Radioact..

[118] Chen C., Sinclair L., Fortin R., Coyle M., Samson C. (2020). In-flight performance of the Advanced Radiation Detector for UAV Operations (ARDUO). Nucl. Instrum. Methods Phys. Res. Sect. A Accel. Spectrometers Detect. Assoc. Equip..

[119] Baena-Navarro R., Torres-Hoyos F. (2020). Design and assembly of an IoT-based device to determine the absorbed dose of gamma and UV radiation. Appl. Radiat. Isot..

[120] Molnar A., Stojcsics D., Lovas I., Domozi Z. Gamma radiation distribution map creation using a small-sized drone. Proceedings of the 2018 IEEE 18th International Symposium on Computational Intelligence and Informatics (CINTI).

[121] Morita T., Oyama K., Mikoshi T., Nishizono T. Decision making support of UAV path planning for efficient sensing in radiation dose mapping. Proceedings of the 2018 IEEE 42nd Annual Computer Software and Applications Conference (COMPSAC).

[122] Abbaraju P., Voyles R. Sensing and sampling of trace contaminations by a dexterous hexrotor UAV at nuclear facilities-18600. Proceedings of the WM2018 Symposium Conference.

[123] Briechle S. (2020). Detection of radioactive waste sites in the Chornobyl exclusion zone using UAV-based lidar data and multi-spectral imagery. ISPRS J. Photogramm. Remote Sens..

[124] Briechle S., Sizov A., Tretyak O., Antropov V., Molitor N., Krzystek P. (2018). Uav-Based Detection of Unknown Radioactive Biomass Deposits in Chernobyl’s Exclusion Zone. ISPRS Int. Arch. Photogramm. Remote. Sens. Spat. Inf. Sci..

[125] Fesenko H., Kliushnikov I. (2020). NPP Monitoring Missions via a Multi-Fleet of Drones: Reliability Issues. Cyber Security and Safety of Nuclear Power Plant Instrumentation and Control System.

[126] Torianyk V., Kharchenko V., Zemlianko H. IMECA based assessment of internet of drones systems cyber security considering radio frequency vulnerabilities. Proceedings of the 2nd International Workshop on Intelligent Information Technologies and Systems of Information Security.

[127] Yao J. (2021). Intelligent and Secure Fog-Aided Internet of Drones. Ph.D. Thesis.

[128] Luke P., Amman M., Lee J.S. (2001). A CdZnTe coplanar-grid detector array for environmental remediation. Nucl. Instrum. Methods Phys. Res. Sect. Accel. Spectrometers Detect. Assoc. Equip..

[129] Hossain I., Sharip N., Viswanathan K. (2012). Efficiency and resolution of HPGe and NaI (Tl) detectors using gamma-ray spectroscopy. Sci. Res. Essays.

[130] Diggins Z.J., Mahadevan N., Herbison D., Karsai G., Barth E., Reed R.A., Schrimpf R.D., Weller R.A., Alles M.L., Witulski A. (2014). Range-Finding Sensor Degradation in Gamma Radiation Environments. IEEE Sens. J..

[131] Peterson J., Li W., Cesar-Tondreau B., Bird J., Kochersberger K., Czaja W., McLean M. (2019). Experiments in unmanned aerial vehicle/unmanned ground vehicle radiation search. J. Field Robot..

[132] Nagatani K., Kiribayashi S., Okada Y., Otake K., Yoshida K., Tadokoro S., Nishimura T., Yoshida T., Koyanagi E., Fukushima M. Gamma-ray irradiation test of electric components of rescue mobile robot Quince. Proceedings of the 2011 IEEE International Symposium on Safety, Security, and Rescue Robotics.

[133] FPV Frame Materials. https://www.getfpv.com/learn/fpv-essentials/fpv-frame-materials/.

[134] Swider-Lyons K., Stroman R., Page G., Schuette M., Mackrell J., Rodgers J. (2011). Hydrogen fule cell propulsion for long endurance small UVAs. AIAA Centennial of Naval Aviation Forum "100 Years of Achievement and Progress".

[135] Rajendran P., Smith H. (2018). Development of Design Methodology for a Small Solar-Powered Unmanned Aerial Vehicle. Int. J. Aerosp. Eng..

[136] Zhang J., Campbell J.F., Sweeney D.C., Hupman A.C. (2021). Energy consumption models for delivery drones: A comparison and assessment. Transp. Res. Part D: Transp. Environ..

[137] Koubâa A., Ammar A., Alahdab M., Kanhouch A., Azar A.T. (2020). DeepBrain: Experimental Evaluation of Cloud-Based Computation Offloading and Edge Computing in the Internet-of-Drones for Deep Learning Applications. Sensors.

[138] Nguyen T., Katila R., Nguyen T. (2021). A Novel Internet-of-Drones and Blockchain-based System Architecture for Search and Rescue. arXiv.

[139] Luo S., Li H., Wen Z., Qian B., Morgan G., Longo A., Rana O., Ranjan R. (2021). Blockchain-Based Task Offloading in Drone-Aided Mobile Edge Computing. IEEE Netw..

[140] Belgium Scientists Develops Drones for Radiation Monitoring. https://www.neimagazine.com/news/newsbelgian-scientists-develop-drones-for-radiation-monitoring-8757417.

[141] Marturano F., Ciparisse J.-F., Chierici A., D’Errico F., Di Giovanni D., Fumian F., Rossi R., Martellucci L., Gaudio P., Malizia A. (2020). Enhancing Radiation Detection by Drones through Numerical Fluid Dynamics Simulations. Sensors.

[142] Roh S. (2017). Big Data Analysis of Public Acceptance of Nuclear Power in Korea. Nucl. Eng. Technol..

[143] Fernández-Caramés T.M., Fraga-Lamas P. (2018). A Review on the Use of Blockchain for the Internet of Things. IEEE Access.

[144] Yaacoub J.-P., Noura H., Salman O., Chehab A. (2020). Security analysis of drones systems: Attacks, limitations, and recommendations. Internet Things.

[145] Drone ‘Containing Radiation’ Lands on Roof of Japanese PM’s Office. https://www.theguardian.com/world/2015/apr/22/drone-with-radiation-sign-lands-on-roof-of-japanese-prime-ministers-office.

[146] Shear M.D., Schmidt M.S. (2015). White House Drone Crash Described as a U.S. Worker’s Drunken Lark. The New York Times.

[147] Stöcker C., Bennett R., Nex F., Gerke M., Zevenbergen J. (2017). Review of the Current State of UAV Regulations. Remote Sens..

[148] Malaysia UAS Regulations. https://www.droneregulations.info/Malaysia/MY.html#country-search.

